# *BDNF* Val66Met moderates episodic memory decline and tau biomarker increases in early sporadic Alzheimer’s disease

**DOI:** 10.1093/arclin/acae014

**Published:** 2024-03-07

**Authors:** Diny Thomson, Emily Rosenich, Paul Maruff, Yen Ying Lim, for the Alzheimer’s Disease Neuroimaging Initiative

**Affiliations:** Turner Institute for Brain and Mental Health, School of Psychological Sciences, Monash University, Clayton, VIC 3168, Australia; Cogstate Ltd, Melbourne, VIC 3000, Australia; Turner Institute for Brain and Mental Health, School of Psychological Sciences, Monash University, Clayton, VIC 3168, Australia; Cogstate Ltd, Melbourne, VIC 3000, Australia; Turner Institute for Brain and Mental Health, School of Psychological Sciences, Monash University, Clayton, VIC 3168, Australia

**Keywords:** Alzheimer’s disease, Brain-derived neurotrophic factor, Episodic memory, Cerebrospinal fluid, Tau protein

## Abstract

**Objective:**

Allelic variation in the brain-derived neurotrophic factor (*BDNF*) Val66Met polymorphism has been shown to moderate rates of cognitive decline in preclinical sporadic Alzheimer’s disease (AD; i.e., Aβ + older adults), and pre-symptomatic autosomal dominant Alzheimer’s disease (ADAD). In ADAD, Met66 was also associated with greater increases in CSF levels of total-tau (t-tau) and phosphorylated tau (p-tau_181_). This study sought to determine the extent to which *BDNF* Val66Met is associated with changes in episodic memory and CSF t-tau and p-tau_181_ in Aβ + older adults in early-stage sporadic AD.

**Method:**

Aβ + Met66 carriers (*n* = 94) and Val66 homozygotes (*n* = 192) enrolled in the Alzheimer’s Disease Neuroimaging Initiative who did not meet criteria for AD dementia, and with at least one follow-up neuropsychological and CSF assessment, were included. A series of linear mixed models were conducted to investigate changes in each outcome over an average of 2.8 years, covarying for CSF Aβ_42_, *APOE* ε4 status, sex, age, baseline diagnosis, and years of education.

**Results:**

Aβ + Met66 carriers demonstrated significantly faster memory decline (*d* = 0.33) and significantly greater increases in CSF t-tau (*d* = 0.30) and p-tau_181_ (*d* = 0.29) compared to Val66 homozygotes, despite showing equivalent changes in CSF Aβ_42_.

**Conclusions:**

These findings suggest that reduced neurotrophic support, which is associated with Met66 carriage, may increase vulnerability to Aβ-related tau hyperphosphorylation, neuronal dysfunction, and cognitive decline even prior to the emergence of dementia. Additionally, these findings highlight the need for neuropsychological and clinicopathological models of AD to account for neurotrophic factors and the genes which moderate their expression.

## Introduction

The development and application of biomarkers of beta-amyloid (Aβ) and tau in natural history studies of aging and dementia shows that Alzheimer’s disease (AD) pathology can emerge up to 30 years prior to individuals meeting any clinical criteria for dementia ([Bibr ref42][Bibr ref42], 2013). Careful prospective neuropsychological studies demonstrate that the pre-dementia stage of AD is characterized by a subtle but relentless decline in episodic memory (Collie&Maruff, 2000; [Bibr ref33][Bibr ref33], 2007; [Bibr ref40][Bibr ref40], 2006). Greater precision in neuropsychological models of early AD could therefore provide a basis for the detection and management of the disease in older adults who are not demented but who have elevated levels of AD biomarkers. One method of improving brain-behavior models is to examine how variation in AD biology (e.g., levels of Aβ), demographic characteristics (e.g., sex) or variation in genes (e.g., carriage of the apolipoprotein e4 allele [*APOE* ε4) can influence the memory decline that occurs in early AD ([Bibr ref4][Bibr ref4], 2018; [Bibr ref27][Bibr ref27], 2014b; [Bibr ref26][Bibr ref26], 2017b). There is now strong and well-replicated evidence that allelic variation in the brain-derived neurotrophic factor (*BDNF)* Val66Met (rs6265) polymorphism exerts substantial influence on rates of neurodegeneration and cognitive decline in adults with elevated levels of Aβ (Aβ+) ([Bibr ref2][Bibr ref2], 2017; [Bibr ref15][Bibr ref15], 2021; [Bibr ref28][Bibr ref28], 2013; [Bibr ref25][Bibr ref25], 2014a; [Bibr ref29][Bibr ref29], 2015; [Bibr ref21][Bibr ref21], 2016; [Bibr ref20][Bibr ref20], 2017a; [Bibr ref22][Bibr ref22], 2018; [Bibr ref23][Bibr ref23], 2021; [Bibr ref24][Bibr ref24], 2022; [Bibr ref3][Bibr ref3][Bibr ref3][Bibr ref3], 2021). Understanding of relationships between *BDNF* Val66Met and changes in episodic memory and AD biomarkers could therefore inform neuropsychological models of early AD.

BDNF is expressed widely in the central nervous system (CNS) and is important for long-term potentiation and synaptic plasticity. Approximately 30% of the population carry the *BDNF* Met66 allele ([Bibr ref39][Bibr ref39], 2018), which is associated with reduced activity-dependent secretion of BDNF and specific impairments in hippocampal-dependent encoding and retrieval processes, hippocampal volume, and episodic memory in both cognitively normal samples ([Bibr ref10][Bibr ref10], 2003; [Bibr ref1e][Bibr ref1e], 2010; [Bibr ref17][Bibr ref17], 2003) and across the spectrum of AD. For example, when compared to compared to matched Aβ + Val66 homozygotes, non-demented Aβ + older adults who carry the Met66 allele have shown an ~ 18% greater episodic memory decline over 3–10 years ([Bibr ref2][Bibr ref2], 2017; [Bibr ref28][Bibr ref28], 2013; [Bibr ref25][Bibr ref25], 2014a; [Bibr ref23][Bibr ref23], 2021; [Bibr ref3][Bibr ref3][Bibr ref3][Bibr ref3], 2021) and an ~ 12% greater decrease in hippocampal volume over 3 years ([Bibr ref28][Bibr ref28], 2013; [Bibr ref25][Bibr ref25], 2014a), despite showing equivalent rates of Aβ accumulation. Non-demented Aβ + older adult Met66 carriers have also shown faster declines in other neuropsychological domains (e.g., executive function, language) across 3 years compared to Val66 homozygotes ([Bibr ref28][Bibr ref28], 2013). In pre-symptomatic autosomal dominant Alzheimer’s disease (ADAD), a quantitatively similar acceleration of Aβ + related neurodegeneration and cognitive decline also occurred in those who also carried the Met66 allele ([Bibr ref22][Bibr ref22], 2018), again, with equivalent decreases in levels of soluble Aβ_42_ in CSF ([Bibr ref22][Bibr ref22], 2018; [Bibr ref24][Bibr ref24], 2022). Further exploration of these relationships in ADAD indicated that Met66 carriage was also associated with a ~ 41% increase in CSF total tau (t-tau) and tau phosphorylated at threonine 181 (p-tau_181_) over three years ([Bibr ref22][Bibr ref22], 2018).

In humans and animals, carriage of a Met66 allele is associated with reduced CNS levels of BDNF ([Bibr ref10][Bibr ref10], 2003). Hence, in pre-symptomatic ADAD, reduced CNS BDNF may allow the faster accumulation of tau and a consequent acceleration of symptoms ([Bibr ref22][Bibr ref22], 2018; [Bibr ref24][Bibr ref24], 2022). This hypothesis accords with data from *in vitro* studies showing that reduction of BDNF in AD is specific to tangle-bearing neurons ([Bibr ref13][Bibr ref13], 1999; [Bibr ref31][Bibr ref31], 1999), and with animal and post-mortem studies showing that the extent of BDNF reduction in the hippocampus was related to the magnitude of cognitive impairment ([Bibr ref8][Bibr ref8], 1997; [Bibr ref10][Bibr ref10], 2003). However, it is possible that the relationship between Met66 carriage and higher tau levels observed in ADAD was a consequence of their younger age, more aggressive form of AD, or their carriage of mutations in *PSEN*-1, *PSEN*-2, or *APP* genes. Therefore, to understand the importance of BDNF in AD, it is necessary to determine whether *BDNF* Met66 carriage is related to increases in tau and memory decline in the pre-dementia stages of sporadic AD.

The aim of this study was to determine the extent to which Met66 carriage is related to rates of change in episodic memory, and in CSF t-tau, p-tau_181_, and Aβ_42_, in Aβ + older adults who do not meet clinical criteria for dementia. The hypothesis was that non-demented Aβ + Met66 carriers would show greater decline in episodic memory, and faster increases in CSF t-tau and p-tau_181_ when compared to Val66 homozygotes, despite showing equivalent changes in CSF Aβ_42_.

## Method

### Participants

Data used in the preparation of this article were obtained from the Alzheimer’s Disease Neuroimaging Initiative (ADNI) database (adni.loni.usc.edu). The ADNI was launched in 2003 as a public-private partnership, led by Principal Investigator Michael W. Weiner, MD. The primary goal of ADNI has been to test whether serial magnetic resonance imaging, positron emission tomography (PET), other biological markers, and clinical and neuropsychological assessment can be combined to measure the progression of mild cognitive impairment (MCI) and early AD. The ADNI study has consisted of four phases: ADNI-1, ADNI-GO, ADNI-2, and, most recently, ADNI-3. For up-to-date information, see www.adni-info.org.

Recruitment processes and inclusion/exclusion criteria for ADNI have been described in detail previously ([Bibr ref32][Bibr ref32], 2010). Broadly, participants were included in ADNI if they were aged 55–90 years and did not have any significant physical, psychiatric or neurological disorders other than AD. At study entry, participants could be cognitively normal or meet clinical criteria for MCI or AD. Cognitive normality was determined by the absence of cognitive complaints, a Mini-Mental State Examination (MMSE) score of 24–30, a CDR score of 0, and a score of ≥9 (i.e., 16 years of education) or ≥ 5 (e.g., 8–15 years of education) on the Logical Memory II (Delayed Recall) subscale. MCI was distinguished by an MMSE score of 24–30 and a CDR score of 0.5 with the memory box score being ≥0.5. AD was further distinguished by a CDR of 0.5 or 1. Diagnoses of MCI and AD required scores of ≤8 (i.e., 16 years of education), ≤4 (8–15 years of education), or ≤ 2 (i.e., 0–7 years of education) on the Logical Memory II (Delayed Recall) subscale ([Bibr ref32][Bibr ref32], 2010).

ADNI participants were included in the current study if they had provided CSF samples and completed neuropsychological assessments on at least two assessment timepoints, were classified as CSF Aβ+, and did not meet clinical criteria for AD dementia during the visit closest to their first lumbar puncture. Only ADNI-1, ADNI-2, and ADNI-GO participants were included in this study.

#### Genetics

Genotype data for the *BDNF* Val66Met (rs6265) polymorphism was extracted using PLINK, an open-source program for analysing whole genome data ([Bibr ref34][Bibr ref34], 2007). Genetic polymorphisms were not used diagnostically. Genotype data were cleaned by applying a minimum call rate for single-nucleotide variations (SNVs, formerly SNPs) and individuals (98%); SNVs not in Hardy–Weinberg equilibrium (*p* < 1 × 10–6) were excluded. No SNVs were removed because of low minor allele frequency. *BDNF* genotype was blind to all neuropsychological raters.

#### CSF biomarkers

Fasted CSF samples were collected via lumbar puncture on the morning of assessments, following procedures described previously ([Bibr ref38][Bibr ref38], 2009). In summary, CSF concentrations CSF t-tau, CSF p-tau_181_ and Aβ_42_ were measured using Luminex bead-based multiplexed xMAP technology immunoassay (INNO-BIA Alzbio3; Innogenetics). All samples were shipped on dry ice to the ADNI Biomarker Core laboratory at the University of Pennsylvania Medical Center. Aβ + was classified when CSF Aβ_42_ levels were below the previously validated cutpoint value of 980 pg/mL ([Bibr ref16][Bibr ref16], 2018).

#### ADNI memory composite

Detailed methods for deriving the ADNI memory (ADNI-Mem) composite have been described previously ([Bibr ref9][Bibr ref9], 2012). Briefly, the ADNI-Mem composite is derived from a longitudinal single factor model that incorporates existing verbal episodic memory measures from the ADNI neuropsychological battery: The Rey Auditory Verbal Learning Test (RAVLT; Schmidt, 1996), the Alzheimer’s Disease Assessment Scale-Cognitive Subscale (ADAS-Cog; [Bibr ref36][Bibr ref36], 1984), three-word recall from the MMSE ([Bibr ref14][Bibr ref14], 1975), and the Logical Memory subtest of the Wechsler Memory Scale. The composite score was originally standardized based on a sample of 803 eligible ADNI participants with longitudinal cognitive data available. It has a mean of 0 and variance of 1 ([Bibr ref9][Bibr ref9], 2012). Use of the composite score, as opposed to individual test scores, can circumvent challenges associated with variability in ADNI’s neuropsychological test battery (e.g., throughout the course of ADNI, two versions of the RAVLT and three versions of the ADAS-Cog were used), changes in tests used over time, and in handling missing data ([Bibr ref9][Bibr ref9], 2012).

### Standard protocol approvals, registrations, and patient consent

Institutional review boards of all participating institutions of ADNI provided approval for the study. All participants at each site provided written informed consent prior to the commencement of any study procedures.

### Data analysis

Analyses were performed in R v.4.1.3. using the following packages: “ggplot2”, “psych”, “gmodels”, “lme4”, “lmerTest”, “emmeans”, “effects”, “dplyr”, “multcomp”, “Hmisc”, “cowplot”, “tidyr”, “car”, “stringr”, “reshape2” and “lubridate”.

Baseline demographic differences between Met66 carriers and Val66 homozygotes were assessed using linear regressions for continuous variables and chi-squared tests for categorical variables. “Baseline” was defined as the first visit at which participants’ CSF samples were collected. An independent samples *t*-test was also conducted to assess differences in baseline CSF Aβ_42_ levels. For longitudinal outcomes of interest (episodic memory, CSF t-tau, CSF p-tau_181_,) a series of analyses of covariance (ANCOVA) were conducted with *APOE* ε4 status, sex, age, baseline diagnosis, years of education and CSF Aβ_42_ levels were entered as covariates. To further explore episodic memory performance on the ADNI-Mem, an ANCOVA was also conducted using data derived from participants’ final visit. A change score (i.e., between baseline visit and final visit) was extracted by subtracting scores at baseline from scores at follow-up.

To test the hypothesis that Aβ + Met66 carriers would show faster declines in episodic memory and greater increases in CSF t-tau and p-tau_181_ compared to Val66 homozygotes, despite showing equivalent changes in CSF Aβ_42_, a series of linear mixed effects models (LMM) were conducted for each outcome variable (i.e., episodic memory, CSF t-tau, CSF p-tau_181_, CSF Aβ_42_,). LMMs were chosen due to their robustness to missing data in a longitudinal design, ability to account for individual variability in change across time, and as they account for fixed and random effects (Verbeke, 1997).

Firstly, to investigate the extent to which Met66 carriage influenced change in Aβ over time compared to Val66 homozygosity, CSF Aβ_42_ data was included as the dependent variable in an LMM including *APOE* e4 status, sex, age, baseline diagnosis and years of education as covariates. “Participant” was entered as a random effect. Then, to examine the extent to which Met66 carriage influenced change in episodic memory, CSF t-tau, CSF p-tau_181_ and CSF Aβ_42_ over time compared to Val66 homozygosity, data for each of these outcomes were included in a series of equivalent LMMs, with baseline CSF Aβ_42_ as an additional covariate. Means (and standard errors, SE) of slopes were extracted to calculate the magnitude of difference in the rate of change in each biomarker and cognitive outcome between Met66 carriers and Val66 homozygotes, measured using Cohen’s *d.* Small (<0.20), medium (0.30–0.70) and large (>0.80) effect sizes were interpreted according to established convention (Cohen, 2016).

## Results

### Demographic and clinical characteristics

A total of 286 participants (94 Met66 carriers, 192 Val66 homozygotes) from ADNI-1, ADNI-GO and ADNI-2 met criteria for inclusion in the analysis. Table 1 summarizes the demographic and clinical characteristics of Aβ + Met66 carriers and Val66 homozygotes at baseline. Groups did not differ significantly on any clinical or demographic measure, except for their baseline scores on the ADNI-Mem, where Met66 carriers showed significantly poorer performance, of a moderate magnitude, compared to Val66 homozygotes (*d* = 0.30). On average, participants were followed for 2.8 years (*SD* = 1.82, *range* = 0.70–10.21), including an average of 2.71 assessments (*SD* = 1.13, *range* = 2–7).

**Table 1:** Baseline demographic, clinical and biological characteristics of Aβ + non-demented Met66 carriers and Val66 homozygotes

	**Met66 carriers**	**Val66 homozygotes**		**Total Sample**
	*n* = 94	*n* = 192		*n* = 286
	***n* (%)**	***n* (%)**	***p***	***n* (%)**
*N* (%) female	32 (34.04%)	83 (43.23%)	0.137	115 (40.21%)
*N* (%) *APOE* ε4	54 (57.45%)	110 (57.29%)	0.943	164 (57.34%)
*N* (%) MCI	69 (73.40%)	135 (70.31%)	0.587	204 (71.33%)
	**Mean (SD)**	**Mean (SD)**	***p***	**Mean (SD)**
Baseline age (years)	74.19 (6.59)	73.48 (6.39)	0.385	73.71 (6.46)
Education (years)	16.36 (2.59)	16.07 (2.79)	0.401	16.17 (2.72)
GDS	1.45 (1.30)	1.47 (1.38)	0.899	1.46 (1.35)
CDR	0.37 (0.23)	0.35 (0.23)	0.442	0.36 (0.23)
CDR-SOB	1.24 (1.18)	1.12 (1.03)	0.367	1.16 (1.09)
MMSE	27.63 (1.96)	27.86 (1.86)	0.336	27.78 (1.90)
ADNI-Mem	0.09 (0.75)	0.31 (0.74)	**0.017**	0.20 (0.75)
CSF t-tau (pg/mL)	315.71 (141.2)	294.46 (125.11)	0.197	305.09 (133.16)
CSF p-tau_181_ (pg/mL)	31.36 (15.97)	29.20 (14.03)	0.244	30.28 (15.00)
CSF Aβ42	688.622 (215.24)	710.89 (197.21)	0.389	699.76 (206.23)

*Note*: Bold values emphasize statistical significance at *p* < 0.05.

*Abbreviations.* ADNI-Mem = ADNI memory composite; *APOE* ε4 = apolipoprotein E; MCI = mild cognitive impairment; SD = standard deviation; GDS = Geriatric Depression Scale; CDR = Clinical Dementia Rating Scale; CDR-SOB = Clinical Dementia Rating Scale Sum of Boxes; MMSE = Mini-Mental State Examination; CSF = cerebrospinal fluid; t-tau = total tau; p-tau_181_ = phosphorylated tau at threonine 181; ADNI = Alzheimer’s Disease Neuroimaging Initiative.

### Differences over time between Aβ + Met66 carriers and Val66 homozygotes on episodic memory and AD biomarkers

**Fig. 1:**
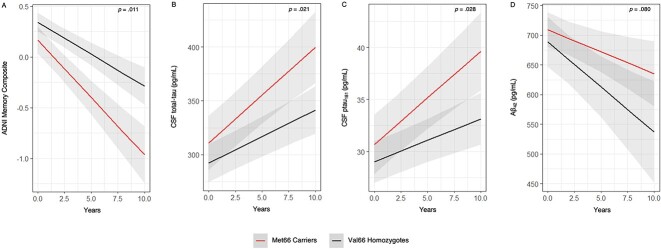
Rates of Change in (A) CSF t-tau, (B) ptau181, (C) Aβ_42_, and (D) Episodic Memory (Measured by the ADNI Memory Composite) Over 10 Years in Non-Demented Older Adult Aβ+ Met66 Carriers and Val66 Homozygotes, Adjusted for CSF Aβ_42_ (Except for the CSF Aβ_42_ Outcome), APOE ε4 Status, Sex, Age, Baseline Diagnosis, and Years of Education.

The results of the LMM indicated a significant *BDNF* × time interaction for episodic memory, whereby Aβ + Met66 carriers showed significantly greater decline in episodic memory, measured by the ADNI-Mem composite, compared to Met66 carriers over an average of 2.8 years (Table 2; Figure 1). The magnitude of this difference was, by convention, moderate (*d* = 0.33). Further examination of this difference indicated that although Met66 carriers performed worse than Val66 homozygotes on the ADNI-Mem composite at baseline (Table 1), Met66 carriers also performed significantly worse than Val66 homozygotes (Table 3) at the final visit, even after accounting for the number of visits. When performance on the ADNI-Mem composite was considered as a change from baseline to final visit, Met66 carriers also showed greater decline than Val66 homozygotes (Table 3). The magnitude of this difference in the rate of episodic decline is equivalent to that in the difference in slopes derived from the LMM.

**Table 2:** Summary of results from the LMMs exploring two-way interactions between BDNF Val66Met × time on episodic memory (measured by ADNI-Mem), CSF t-tau, p-tau_181_ and Aβ_42_, outcomes over 10 years in Aβ + Met66 carriers and Val66 homozygotes

	***BDNF* Val66Met**		**Time**		***BDNF* × time**	
	**Est. (SE)**	***p***	**Est. (SE)**	***p***	**Est. (SE)**	***p***
ADNI-Mem	**0.28 (0.09)**	**<0.001**	**−0.22 (0.03)**	**<0.001**	**0.10 (0.04)**	**0.011**
CSF t-tau (pg/mL)	0.20 (0.12)	0.099	**0.13 (0.02)**	**<0.001**	**−0.05 (0.02)**	**0.021**
CSF p-tau_181_ (pg/mL)	−0.17 (0.12)	0.158	**0.11 (0.02)**	**<0.001**	**−0.05 (0.02)**	**0.028**
CSF Aβ_42_ (pg/mL)	0.15 (0.11)	0.175	**−0.13 (0.04)**	**<0.001**	0.07 (0.04)	0.080

*Note:* Bold values emphasize statistical significance at *p* < 0.05; Covariates: CSF Aβ_42_ (except for CSF Aβ_42_ outcome), *APOE* ε4 status, sex, age, baseline diagnosis, years of education.

*Abbreviations.* ADNI-Mem = ADNI memory composite; *BDNF* = brain-derived neurotrophic factor; Est. = estimate, SE = standard error; CSF = cerebrospinal fluid; t-tau = total tau; p-tau_181_ = phosphorylated tau at threonine 181; Aβ_42_ = amyloid beta peptide 42; ADNI = Alzheimer’s Disease Neuroimaging Initiative; pg/mL = picograms per milliliter.

**Table 3:** Summary of Met66 carriers’ and Val66 homozygotes’ memory performance on the ADNI-Mem at the final visit, including change between baseline and final visit and the modelled slope derived from the linear mixed model

	**Final visit**	**Raw change scores**	**LMM slope**
	**Mean (SD)**	**Mean (SD)**	**Mean (SD)**
**Met66 carriers**	0.03 (0.83)	−0.29 (0.54)	−0.22 (0.31)
**Val66 homozygotes**	0.39 (0.85)	−0.11 (0.55)	−0.13 (0.28)
**Cohen’s *d* (95%CI)**	0.43 (0.17, 0.67)	0.33 (0.08, 0.58)	0.33 (0.08, 0.58)

*Note*: SD = standard deviation; CI = confidence interval; LMM = linear mixed model

The LMMs also indicated a significant *BDNF* × time interaction for CSF t-tau and p-tau_181_, whereby Aβ + Met66 carriers showed significantly greater increases in CSF t-tau (*d* = 0.30) and p-tau_181_ (*d* = 0.29) compared to Val66 homozygotes over an average of 2.8 years (Table 2; Figure 1). The magnitude of these differences in rates of change were, by convention, moderate. Met66 carriers did not show a statistically greater decrease in CSF Aβ_42_ compared to Val66 homozygotes, with the magnitude of this difference small (*d* = 0.23).

## Discussion

Supporting our hypothesis, the *BDNF* Val66Met polymorphism exerted a substantial influence on the rate of decline in episodic memory and increases in CSF levels of t-tau and p-tau_181_ over an average of 2.8 years. Despite showing equivalent changes in CSF Aβ_42_, non-demented Aβ + Met66 carriers showed not only poorer memory performance at baseline compared to Val66 homozygotes (*d* = 0.30), but also showed faster memory decline compared to Val66 homozygotes (~15%). Similarly, Aβ + Met66 carriers showed faster increases in CSF t-tau (~12%) and p-tau_181_ (~15%) compared to Val66 homozygotes. These findings are consistent with observations made in ADAD over the same time period, where Met66 was also associated with an ~ 29% greater decline in episodic memory, and ~ 41% greater increase in CSF t-tau and p-tau_181_ when compared to ADAD Val66 homozygotes ([Bibr ref22][Bibr ref22], 2018). These results further support the hypothesis that *BDNF* Val66Met moderates the effects of Aβ on AD clinical disease progression via its effect on tau ([Bibr ref22][Bibr ref22], 2018; [Bibr ref24][Bibr ref24], 2022) by showing this effect on tau in the sporadic form of AD. Despite Aβ + being the hallmark characteristic of AD, younger Aβ- Met66 carriers do not show any change in CSF Aβ_42_, CSF tau, brain volume or cognition ([Bibr ref22][Bibr ref22], 2018). Therefore, these findings show that in non-demented Aβ + older adults, variation in the *BDNF* Val66Met polymorphism has a clinically important influence on increases in tau, neurodegeneration, and memory decline.

The magnitude of difference in the rate of episodic memory decline between groups is consistent with previous observations in cross-sectional and longitudinal studies in the preclinical ([Bibr ref2][Bibr ref2], 2017; [Bibr ref28][Bibr ref28], 2013) and prodromal ([Bibr ref25][Bibr ref25], 2014a; [Bibr ref22][Bibr ref22], 2018) stages of sporadic AD, as well as in pre-symptomatic ADAD ([Bibr ref21][Bibr ref21], 2016; [Bibr ref23][Bibr ref23], 2021; [Bibr ref24][Bibr ref24], 2022). Although hippocampal volume was not measured in this study, previous studies show that hippocampal atrophy is~14% faster in Aβ + Met66 carriers compared to Val66 homozygotes across preclinical and prodromal sporadic AD ([Bibr ref28][Bibr ref28], 2013; [Bibr ref25][Bibr ref25], 2014a) and pre-symptomatic ADAD ([Bibr ref22][Bibr ref22], 2018). A recent study conducted in preclinical, prodromal, and clinical stages of AD dementia, conducted over 12.5 years, suggested that the nature of memory decline in Met66 carriers may change with disease severity. Specifically, although Met66 carriage was associated with faster memory decline in preclinical and prodromal AD, in the clinical stages of AD dementia, memory decline is only evident in Val66 homozygotes, as memory performance in Met66 carriers had reached the lowest possible values (i.e., a floor effect; [Bibr ref23][Bibr ref23], 2021). This likely reflects that any protective effects conferred by Val66 homozygosity on synaptic plasticity and neuronal survival become limited once individuals progress to AD dementia. The sample of Aβ + individuals with clinical AD dementia, *BDNF* genotyping, and CSF biomarker outcomes in ADNI was not sufficiently large (*n* = 64; 36% Met carriers) to test this interaction. As such, future studies using larger samples of individuals with AD dementia are necessary to clarify how neurotrophic factors influence tau levels in advanced AD. Additionally, although memory differences in Aβ + Met66 carriers and Val66 homozygotes have been consistently observed, the magnitude of difference in memory decline between groups remains moderate in the magnitude, suggesting that their utility in clinical practice will be limited. However, the results of this study do inform current neuropsychological models of AD by confirming that subtle memory declines are related to accumulation of tau, and that this is hastened when there is reduced expression of growth factors such as BDNF.

Recent advances in understanding of tau biology have allowed the development of models of tau kinetics according to site-specific levels in mass-spectrometry measured tau phosphorylation. It is now agreed that site-specific tau phosphorylation may reflect different clinical stages of AD. For example, CSF tau phosphorylation occupancy at threonine 181 and 217 (p-tau_217_) increases with initial Aβ accumulation, although phosphorylation occupancy at threonine 205 (p-tau_205_) increases only when brain atrophy and clinical symptoms emerge ([Bibr ref1][Bibr ref1], 2020). Recently, we showed that these different levels of tau phosphorylation were also influenced by Met66 carriage. In pre-symptomatic ADAD mutation carriers, Met66 carriers showed greater CSF p-tau_181_ and p-tau_217_ phosphorylation compared to Val66 homozygotes, but equivalent levels of p-tau_205_ phosphorylation ([Bibr ref24][Bibr ref24], 2022). Conversely, in symptomatic mutation carriers, Met66 carriers showed greater levels of CSF t-tau and p-tau_205_ phosphorylation compared to Val66 homozygotes, but equivalent phosphorylation levels of CSF p-tau_181_ and p-tau_217_. The association between Met66 carriage and site-specific tau phosphorylation suggests that Met66 carriers experience greater disease progression in early stages of AD. This is reflected by greater initial increases in p-tau_181_ and p-tau_217_ phosphorylation relative to Val66 homozygotes, and followed by greater increases in t-tau and p-tau_205_ phosphorylation as neuronal dysfunction increases ([Bibr ref1][Bibr ref1], 2020). Measures of p-tau_217_ and p-tau_205_ are not yet available for the ADNI sample. Future studies are needed to confirm the hypothesis that changes in levels of p-tau_217_ and p-tau_205_ isoforms reflect different stages of clinical disease severity in sporadic AD.

The processes by which *BDNF* Val66Met influences tau hyperphosphorylation and episodic memory decline, even prior neurofibrillary tangle formation, has not been fully elucidated. Initially, research in animal and cellular models postulated that tau was responsible for downregulating BDNF expression ([Bibr ref35][Bibr ref35], 2016). However, if downregulation of BDNF by tau was the driver of cognitive decline and neurodegeneration observed in clinical studies, human Met66 carriers and Val66 homozygotes should show equivalent increases in CSF tau in early AD. This was not observed in the current or previous studies ([Bibr ref22][Bibr ref22], 2018). On the contrary, the clinical data suggest that as *BDNF* Met66 reduces BDNF availability in the CNS, this reduction in neurotrophic factors may allow faster Aβ + related tau hyperphosphorylation, subsequent neurodegeneration and cognitive decline. Animal studies do show that loss of BDNF may mediate neurotoxicity of tau downstream of abnormal increases in Aβ ([Bibr ref35][Bibr ref35], 2016). Lower levels of circulating BDNF can also antagonize the major receptor site for BDNF, tropomyosin receptor kinase B (TrkB), which can precipitate rapid tau hyperphosphorylation and subsequent synaptic dysfunction and neuronal degeneration ([Bibr ref11][Bibr ref11], 2005; [Bibr ref44][Bibr ref44], 2019). Additionally, *BDNF* Val66Met may moderate neuroinflammatory responses that affect vulnerability to the downstream effects of Aβ. In vitro studies show that astrocytes can quickly increase expression of BDNF in response to increasing levels of Aβ ([Bibr ref19][Bibr ref19], 2006), which may be an attempt to protect neurons against AD pathogenesis ([Bibr ref12][Bibr ref12], 2014). Thus, one possible integration of these observations is that lower BDNF availability associated with Met66 carriage could reduce resilience to inflammatory processes in early disease stages.

The current finding that *BDNF* Val66Met influences increases in tau in Aβ + older adults, which is consistent with previous findings across different cohorts with varying disease severity and Aβ aetiology, have important implications for the field. First, the effect of the Met66 allele on cognitive decline, at least in the pre-dementia stages of the disease, is substantial [~8%–~40% in preclinical sporadic AD studies ([Bibr ref2][Bibr ref2], 2017; [Bibr ref28][Bibr ref28], 2013; [Bibr ref23][Bibr ref23], 2021; [Bibr ref3][Bibr ref3][Bibr ref3][Bibr ref3], 2021); ~6%–21% in prodromal sporadic AD studies ([Bibr ref25][Bibr ref25], 2014a; [Bibr ref23][Bibr ref23], 2021); ~29% in pre-symptomatic ADAD ([Bibr ref22][Bibr ref22], 2018)], and, therefore, understanding of this effect should inform AD clinicopathological models. Second, the Met66 allele is very common (e.g., 33% of the white population), and given the centrality of Aβ and tau models in AD and in the development of pharmacotherapeutics, the potential influence of variation in *BDNF* Val66Met on study outcomes should be considered. Third, given the frequency of the polymorphism, the magnitude of its effects on tau and cognitive outcomes, and the absence of effects on Aβ, variation on the *BDNF* Val66Met polymorphism could provide a useful clinical tool for challenging knowledge about the extent to which other fluid or imaging biomarkers are associated with Aβ or tau accumulation. For example, examination of the effect of *BDNF* Val66Met on markers of neuroinflammation or synaptic function may further clarify its role in AD clinical progression. Finally, these findings suggest that loss of BDNF that occurs downstream of abnormal increases in Aβ may increase vulnerability to neuronal dysfunction via tau and precipitate accelerated clinical disease progression in pre-dementia stages of AD. Conversely, these findings also suggest that greater BDNF availability can protect neurons, as well as the cognitive functions that depend on them, from Aβ-related cell death. Thus, pharmacologically increasing neurotrophic support in the early stages of AD may be a potential therapeutic target to delay the clinical manifestation of AD dementia ([Bibr ref30][Bibr ref30], 2013). For example, in vitro studies suggest that agonist antibodies (e.g., AS86) that target TrkB promote synaptic growth and repair ([Bibr ref43][Bibr ref43], 2020). Studies in rodents also suggest that TrkB receptor agonists such as 7,8-dihydroxyflavone (7,8-DHF) can block delta-secretase activation, attenuate AD pathology, and reduces cognitive dysfunction ([Bibr ref5][Bibr ref5], 2021). It is important to note, however, that despite the promising therapeutic potential of these TrkB agonists, their safety, and efficacy in humans remains unclear.

Some limitations are noted which restrict the generalizability of the current results. First, although the maximum follow-up time was 10 years, the average follow-up time was only 2.8 years. This limited the ability to investigate the role of *BDNF* Val66Met on clinical disease progression. Second, cognitively normal and MCI participants were combined to enable examination across a more well-powered sample of Aβ + older adults. Although baseline diagnosis was controlled statistically in the statistical modelling, it will be important for future studies with larger sample sizes to clarify the role of *BDNF* Val66Met on changes in tau and memory across the preclinical, prodromal, and dementia stages of AD. Alternatively, harmonization of multiple prospective cohort studies may enable a sufficiently powered study of the effects of *BDNF* Val66Met on disease progression at each stage of AD. This would allow for more detailed investigations into whether *BDNF* Val66Met’s effects on CSF tau biomarkers differ across populations and whether the pattern of memory decline shown in Met66 carriers can be characterized by impairments in specific components of memory across the disease course of AD (e.g., delayed recall, recognition). Given that the Met66 allele is present in ~30% of the white population ([Bibr ref39][Bibr ref39], 2018), a further benefit of expanding the sample to include cohorts is the ability to explore the role of *BDNF* Val66Met across various racial and ethnic groups. This would be informative as the prevalence of the Met66 allele varies across populations (e.g., approximately 70% of individuals from Asian populations carry the Met66 allele; [Bibr ref39][Bibr ref39], 2018). A third limitation of the study is that CSF measures of Aβ_42_ can be susceptible to variability in pre-analytical handling of CSF samples ([Bibr ref1f][Bibr ref1f], 2015). There is also variability in handling of CSF samples across ADNI cohorts. As such, there has been movement towards using a ratio of Aβ_42_/Aβ_40_ for a more sensitive classification of Aβ positivity. Although this ratio was not available for all participants in the current study, it will be important for studies to test this in the future. Finally, although CSF markers of tau provide a single value reflecting neuronal death and neurofibrillary tangle formation, they cannot determine the topography of tau pathology in the brain ([Bibr ref18][Bibr ref18], 2016). It will thus be important for future studies to examine whether changes in tau PET are similar to the changes in CSF tau and p-tau_181_ observed in this study.

These limitations notwithstanding, the results of this study provide support for the hypothesis that *BDNF* Val66Met moderates downstream effects of Aβ + on tau and memory decline in preclinical and prodromal sporadic AD. This study also illuminates that brain areas necessary for episodic memory are dependent on growth factors, such as BDNF, and that factors which compromise the availability of BDNF, such as *BDNF* Met66 carriage, give way to increases in CSF tau and accelerated episodic memory decline. Consistent with other studies in Aβ + older adults at risk of sporadic AD and adults with pre-symptomatic ADAD, subtle episodic memory declines can be detected in the long pre-dementia phase of AD, even prior to meeting clinical criteria for dementia. Taken together, these data show that memory loss in Aβ + adults is related to loss of neuronal function, and that sufficient availability of neuronal growth factors may forestall tau hyperphosphorylation, accelerated neuronal dysfunction and memory decline in the pre-dementia stages of sporadic AD. Given the long pre-dementia stage of AD, these findings highlight the opportunity for more precise detection and management of memory decline in older adults who present with abnormal AD biomarkers and risk factors for reduced growth factor availability.

## Data Availability

Genetic, biomarker and clinical data from ADNI are publicly accessible and are available through a formal application on http://adni.loni.usc.edu.
